# Catalpol Enhances Random-Pattern Skin Flap Survival by Activating SIRT1-Mediated Enhancement of Autophagy

**DOI:** 10.1155/2022/5668226

**Published:** 2022-05-17

**Authors:** Ren-hao Jiang, Cheng-ji Dong, Zhu-liu Chen, Sheng Cheng, Jian-xin Yang, Wei-yang Gao

**Affiliations:** ^1^Department of Orthopaedics, The Second Affiliated Hospital and Yuying Children's Hospital of Wenzhou Medical University, Wenzhou 325027, China; ^2^Zhejiang Provincial Key Laboratory of Orthopaedics, Wenzhou 325027, China

## Abstract

Random-pattern skin flap necrosis limits its application in the clinic. It is still a challenge for plastic surgeons. Catalpol is an effective ingredient extracted from Rehmannia glutinosa, which is reported to promote angiogenesis and protect against ischemic cerebral disease. The aim of our experiment is to assess whether catalpol can facilitate random flap survival and the underlying mechanisms. Male “McFarlane flap” rat models were employed to explore the protective effects of catalpol. The range of necrosis in the flap was calculated 7 days after the models were established. The flap specimens were harvested for further experiments, including angiogenesis, apoptosis, oxidative stress, and autophagy evaluation. Catalpol-treated group promoted the average survival area of the flap than that in the control group. Based on immunohistochemical staining, Western blotting, and ROS detection, we found that catalpol significantly reduces oxidative stress and apoptosis and increases angiogenesis. Hematoxylin and eosin (H&E) staining and laser Doppler images further clarified the enhancement of angiogenesis after catalpol treatment. The impact of catalpol in flap was switched by using 3-methyladenine (3MA), proving the important role of autophagy in curative effect of catalpol on skin flaps. Importantly, the ability of catalpol to regulate autophagy is mediated by the activation of sirtuin 1 (SIRT1) based on its high affinity for SIRT1. Our findings revealed that catalpol improved the viability of random skin flaps by activating SIRT1-mediated autophagy pathway.

## 1. Introduction

Random-pattern skin flaps are universally used for soft tissue defect repair in reconstructive surgery resulting from trauma, cancer excisions, and complications of diabetes [1, 2]. However, what perplexes doctors is the necrosis of skin flap after surgery. Previous studies have reported that the incidence of skin flap necrosis is 10%~15% [3]. Random flap is restricted by the ratio of length to width, generally no more than 2 : 1, which greatly limits its clinical application [4]. The main cause of flap necrosis is blood supply disorder. Therefore, it is necessary to find better drugs and methods to improve the blood supply of the flap and promote the vascular regeneration of the flap as soon as possible. Ischemia-reperfusion (I/R) injury resulted by reperfusion and restoration of blood supply after vascular regeneration can accumulate reactive oxygen species (ROS), leading to cell death [5]. Based on this mechanism, inhibition of oxidative stress and enhancement of angiogenesis have been investigated as possible treatment options.

Sirtuin 1 (SIRT1), a NAD+-dependent class III histone deacetylase, is involved in modulating angiogenesis, apoptosis, oxidative stress, and autophagy [6–8]. Thus far, loss of SIRT1 has been found to aggravate ischemia/reperfusion injury in aged livers [9]. In addition, activation of SIRT1 by Ginsenoside Rc (one of the bioactive components of Panax ginseng) can enhance cardio- and neuroprotective functions under ischemia/reperfusion (I/R) damage conditions [10]. Furthermore, studies have found that increasing SIRT1-dependent autophagy by FGF21 can effectively promote the progression of wound healing [11]. However, the mechanism of regulation of SIRT1 in random flap survival remains unclear.

Autophagy acts as a cytoprotective agent through scavenging damaged cytosolic material-dependent lysosomal system [12]. Increasing autophagy can reduce oxidative stress effects in the vascular system and elevate endothelial cell function in old mice [13]. Autophagy enhanced the angiogenesis activity of human umbilical vein endothelial cell (HUVEC) exposure to high-glucose concentration by reducing cell damage and significantly alleviating oxidative stress [14]. Therefore, autophagy may have a benefit impact on oxidative stress-mediated damage caused by ischemic skin flap necrosis. However, autophagy may accelerate cell death under different circumstances. The correlation between autophagy and random flap necrosis needs more research.

Catalpol (CAT), the main and biological active component of the traditional Chinese herbal medicine Rehmannia glutinosa, was reported to have benefit effects on reducing oxidative stress, promoting angiogenesis and inhibiting apoptosis [15–17]. Recent study has shown that CAT exerts osteogenesis-angiogenesis effects, thus accelerating osteoporotic bone repair by activating STAT3 [18]. Furthermore, CAT could exert a protective role by effectively inducing autophagy in a variety of pathological conditions, including liver steatosis, diabetic nephropathy, and cardiovascular diseases [19–21]. Meanwhile, CAT can alleviate nephropathy induced by adriamycin due to its strong affinity for SIRT1 [22]. Based on the above information, we aimed to investigate the effects of CAT on skin flap survival and the detailed biological mechanisms to explore the role of SIRT1 activated by CAT in random flap. The interaction of autophagy and catalpol in ischemic skin flaps needs further research.

## 2. Materials and Methods

### 2.1. Animals

Male Sprague-Dawley rats (200–250 g) were acquired from the Wenzhou Medical University Laboratory Animal Center. All animals were treated with humanity and nurtured with daily water ad libitum and given free access to regular food. Animals were housed individually in an environmentally controlled condition as previously reported [4]. Experimental protocols involving rats were confirmed by the Guide for the Care and Use of Laboratory Animals of the China National Institutes of Health, and the Animal Care and Use Committee of Wenzhou Medical University approved the experiment (wydw2021-0328). Every effort was made to decrease the number of experimental animals and reduce the pain of animals. The experimental groups were designed as follows: rats were divided randomly into four groups: (1) control (vehicle, *n* = 24), (2) catalpol (CAT, *n* = 24), (3) CAT+3MA (*n* = 24), and (4) CAT+EX527 (*n* = 12).

### 2.2. Reagents

Catalpol (98%) was purchased from Solarbio Science & Technology (Beijing, China). 3-Methyladenine was obtained from Solarbio Science & Technology (Beijing, China). The SIRT1 inhibitor EX527 was obtained from Sigma-Aldrich Chemical Company (Milwaukee, WI, USA). Diaminobenzidine (DAB) developer, pentobarbital sodium, and the H&E staining kit were obtained from Solarbio Science & Technology (Beijing, China). In the molecular studies, the following antibodies were used: anti-Cadherin 5 (A02632-2; Boster Biological Technology, Wuhan, China), anti-GAPDH (AP0063; Biogot Technology, Shanghai, China), anti-VEGF (A12303, ABclonal, China), anti-SIRT1, anti-LC3II, anti-Superoxide Dismutase 1 (SOD1), anti-Cathepsin D (CTSD), anti-Caspase 3 (CAPS3), and anti-Heme Oxygenase 1 (HO1) (13161-1-AP, 14600-1-AP, 10269-1-AP, 21327-1-AP, 19677-1-AP, and 27282-1-AP; Proteintech Group, Chicago, IL, USA); the primary antibody anti-Bax, anti-Bcl-2, anti-AMPK, anti-p-AMPK, anti-mTOR, anti-p-mTOR, anti-Cleaved-Caspase 3 (C-CAPS3), and antiendothelial nitric oxide synthase (eNOS) were obtained from Cell Signaling Technology (CST) (2772S, 15071S, 5832S, 2535S, 2983, 5536S, 9664S, and 32027S; Beverly, MA, USA); the primary antibody anti-SQSTM1/p62, anti-CD34 and anti-Matrix Metalloproteinase 9 (MMP9) were obtained from Abcam(ab56416, ab81289, ab283575; Cambridge, UK).

### 2.3. Animal Model and Drug Administration

All rats were treated with sodium pentobarbital 1% (40 mg/kg, intraperitoneal (ip)) for anesthesia before operation. Then, we established the dorsal random-pattern skin flap model in every rat (3 × 9 cm) as previously described [23]. In brief, after removing the dorsal fur, the caudal-based regions were marked and detached skin and subcutaneous tissue from the deep fascia. Subsequently, all known arteries were sacrificed completely. Ultimately, the separated flap was sutured into the original area with 4-0 nonabsorbable silk. The flap was separated into three equal regions: area I (proximal zone), area II (intermediate zone), and area III (distal zone). The therapeutic group was treated with CAT ip injection of 10 mg/kg for consecutive 7 days after operation, while the control group received the same volume of 0.9% saline solution; the CAT+3MA group was treated with 3MA (15 mg/kg/day, ip) 30 min before CAT administration, and the CAT+EX527 group received EX527 (10 mg/kg/day, ip) 30 min before CAT administration. On the 7th day after operation, all rats were euthanized, and flap tissues were collected for follow-up experiments.

### 2.4. Flap Survival Measurement

Flap survival status were noted daily by the experimenter, including flap facade, color, tissue flexibility, texture, and hair condition from postoperative days 1 to 7. All flaps were photographed, and the flap viability was calculated by high-quality photographs on grid paper on days 3 and 7. The percentage of viable area was quantified as follows: (viable area size ÷ whole flap size) × 100%.

### 2.5. Tissue Edema Assessment

We measured tissue water content to evaluate tissue edema, which is a replaceable marker for the degree of flap necrosis. On day 7 after surgery, tissue samples were harvested and weighed to measure the “wet weight.” Next, we placed tissue samples in a 50°C autoclave and weighed daily until the weight remained the same for 2 days, recorded as “dry weight.” Tissue edema was quantified with the following formula: ([wet weight − dry weight] ÷ wet weight) × 100%.

### 2.6. Laser Doppler Imaging

In order to measure hemoperfusion under the flap, a laser Doppler instrument (PIMII; Lisca, Stockholm, Sweden) was employed before the rats were sacrificed on day 7 postoperatively. The images were obtained after scanning the entire flap of the back. Then, we imported images in moor LDI Review software (ver.6.1; Moor Instruments) to assess blood flow of the skin flaps.

### 2.7. Histological Examination

After euthanizing all animals, equal size specimens (*n* = 6, 1 × 1 cm) were randomly gathered from area II of the flap from each group. Each sample was fixed in 4% paraformaldehyde for 24 h and implanted in paraffin wax. Afterwards, the sample was cut as 4 *μ*m thickness tissue sections. A light microscope (Olympus Corp, Tokyo, Japan) was applied to observe the proliferation of granulation tissue, inflammatory infiltration, and tissue edema. The quantity of vascular cross section per unit area (/mm^2^) was counted to assess the level of microvascular density environment.

### 2.8. Immunohistochemical Staining

Six sections of the middle area of the flap from each group were deparaffinized in xylene, and then, graded ethanol baths were used for rehydration. After rehydrating, the specimens were blocked with 3% (*v*/*v*) hydrogen peroxide. Subsequently, 10.2 mM sodium citrate was applied for antigen repair for 30 min at 95°C. Then, the following primary antibodies were used for incubation overnight as 4°C, including CD34 (1 : 100, ab81289, Abcam), Cadherin5 (1 : 100, A02632-2, Boster Biological Technology), VEGF (1 : 300, A12303, ABclonal), CASP3 (1 : 200, 19677-1-AP, Proteintech), SOD1 (1 : 100, 10269-1-AP, Proteintech), and CTSD (1 : 100, 21327-1-AP, Proteintech). Then, samples were treated with HRP-conjugated secondary antibody (SA00001-1, Proteintech), stained by DAB kit, and counterstained with hematoxylin. Lastly, stained sections were visualized under light microscopy using the DP2-TWAN image-acquisition system (Olympus Corp., Tokyo, Japan). Quantification was performed for Cadherin 5, VEGF, SOD1, CASP3, and CTSD expression levels, and the number of CD34-positive blood vessels was enumerated. Measurements were obtained from three random sections, with six random visual fields each.

### 2.9. Immunofluorescence

The process of deparaffinization and rehydration of the tissue specimens is described above. And then, tissue antigen was treated with 10.2 mM sodium citrate buffer to repair. After being blocked with 10% (*v*/*v*) bovine serum for 30 min, slides were incubated with anti-LC3B (1 : 200, 14600-1-AP, Proteintech) and anti-SIRT1 (1 : 200, 13161-1-AP, Proteintech) at 4°C overnight for primary staining, followed by incubation (1 h, 37°C) with secondary antibodies (Alexa Fluor 488 and 647 AffiniPure Goat Anti-Rabbit IgG, BL067A and BL061A, Biosharp) and DAPI staining. Fluorescence microscope (Olympus, Tokyo, Japan) was applied to evaluate the specimen. The percentage of LC3II-positivity and SIRT1-positivity cells was calculated using six random fields from three random sections of each slide in the dermal layer.

### 2.10. Western Blot Analysis

Skin samples (0.5 cm × 0.5 cm) were obtained from area II of the flap on day 7 postoperatively for Western blotting analyses (−80°C storage condition). An equal amount of protein (60 *μ*g) was used for Western blotting. And then, proteins were separated by SDS-PAGE, followed by transferring to polyvinylidene difluoride. After being blocked with 5% (*w*/*v*) nonfat milk (2 h, room temperature), the membranes were incubated with the specific primary antibodies overnight at 4°C, including MMP9 (1 : 1000, ab283575, Abcam), VEGF (1 : 1000, A12303, ABclonal), Cadherin 5 (1 : 1000, A02632-2; Boster Biological Technology), SOD1 (1 : 1000, 10269-1-AP, Proteintech), eNOS (1 : 1000, 32027S, CST), HO1 (1 : 1000, 27282-1-AP, Proteintech), Bax (1 : 1000, 2772S, CST), Bcl-2 (1 : 1000, 15071S, CST), CAPS3 (1 : 1000, 19677-1-AP, Proteintech), Beclin1 (1 : 1000, 11306-1-AP, Proteintech), LC3II (1 : 1000, 14600-1-AP, Proteintech), CTSD (1 : 1000, 21327-1-AP, Proteintech), P62 (1 : 1000, ab56416, Abcam), SIRT1 (1 : 1000, 13161-1-AP, Proteintech), AMPK, p-AMPK, mTOR, and p-mTOR (1 : 1000, 5832S, 2535S, 2983, and 5536S, CST), followed by incubation with the secondary antibodies (Goat Anti-Mouse IgG or Goat Anti-Rabbit IgG, BL001A; BL023A, Biosharp) (2 h, room temperature). The ECL Plus Reagent Kit was applied to detect the protein bands. The quantification of band density was performed using Image Lab 3.0 software.

### 2.11. ROS Detection of Flap Tissue

ROS levels in the flap tissues were detected with tissue ROS Assay Kit (BestBio Company, China). 50 mg of fresh flap tissue was made into homogenate with 1 mL of homogenate buffer A, and then, homogenate was centrifuged at 4°C for 5 minutes. Supernatant (190 *μ*L) was collected and mixed with 10 *μ*L of BBcellProbe™ O13 ROS probe (BestBio, China) in a 96-well plate for 30-minute incubation in the dark. ROS levels were quantified by a multimode microplate reader (Synergy NEO2, BioTek, USA) with an excitation wavelength of 520 nm and emission wavelength of 606 nm. Flap ROS levels were represented as fluorescence intensity/protein concentration (mg).

### 2.12. Statistical Analyses

All statistical analyses were performed by SPSS software (ver. 19, Chicago, IL, USA). Data are generally expressed as means ± SEM. Statistical analysis was performed with two-tailed followed by unpaired *t*-test when comparing two independent groups. A *p* value less than 0.05 was considered statistically significant.

## 3. Results

### 3.1. CAT Enhances Skin Flap Survival and Reduces Tissue Edema

**Figure 1:**
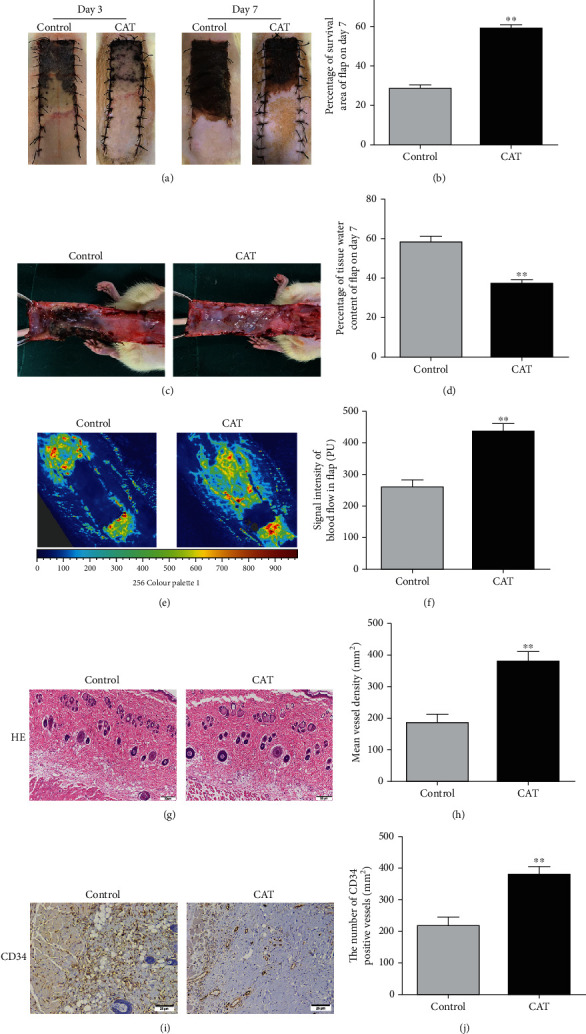
CAT improves the survival of random skin flaps. (a) Dorsal skin flap digital photographs from the control and CAT groups on days 3 and 7, postoperatively. (b) Histogram showing quantification of the percentages of survival area in each group. (c) Images of the inner side of flaps in the control and CAT groups on day 7 postoperatively. (d) Quantitative analysis of percentage of tissue water content in two groups. (e) LDBF images of flaps blood flow intensity in each group on POD 7. (f) Quantitative analysis of blood flow of flaps. (g) The area II of flap tissues H&E staining in the control and CAT groups (200 magnification; scale bar, 50 *μ*m). (h) Quantitative analysis of percentage of MVDs in each group. (i) Images of immunohistochemical staining for CD34 to reveal vessels in the control and CAT groups (200 magnification; scale bar, 25 *μ*m). (j) Quantitative analysis of percentage of CD34-positive vessels in two groups. Data are shown as mean ± SEM, *n* = 6. ^∗^*p* < 0.05 and ^∗∗^*p* < 0.01 vs. the control group.

After the flap model was established, flap survival conditions were observed and recorded. Area III of the flaps became swollen and pale at the early stage. As time went by, the distal area of flaps gradually became black, dry, and stiff, and necrosis gradually appeared. At the later stage, area III displayed total necrosis with tissue becoming darkened, hardened, and shriveled, and some similar changes were observed in area II of flap (Figure 1(a)). On the 7th day, the necrosis boundary was well demarcated in all groups. Here, we found that CAT significantly improved flap survival in the experimental group compared with the control group (Figure 1(b)). Qualitatively, the tissue edema and subcutaneous venous congestion conditions were alleviated by CAT treatment (Figure 1(c)). Moreover, CAT reduced tissue water content (Figure 1(d)), which reflected tissue edema. Hemoperfusion in the CAT group was improved, which was visualized by LDBF (Figure 1(e)), and the signal intensity of blood flow was also significantly superior in CAT-treated group (Figure 1(f)). Microvascular density was also measured by using H&E staining (Figure 1(g)). The vessel density in the CAT group was higher compared to the controls (Figure 1(h)). Meanwhile, IHC showed that the number of CD34-positive vessels was upregulated in the CAT group (Figures 1(i) and 1(j)). On the basis of all above results, we conclude that CAT enhances the survival of skin random flaps.

### 3.2. CAT Improves Angiogenesis

**Figure 2:**
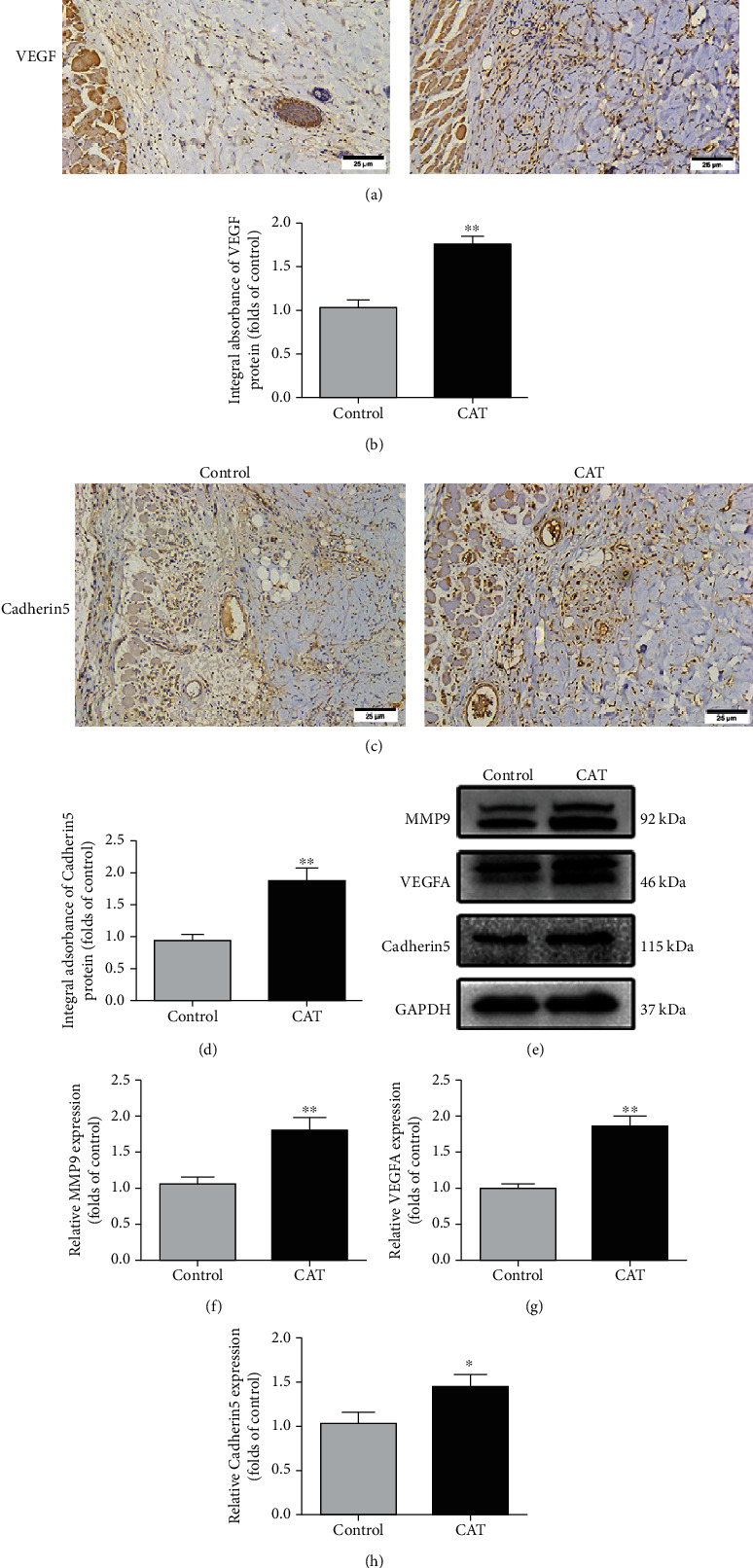
CAT enhances angiogenesis in random skin flaps. (a, c) Immunohistochemical staining of proteins VEGF and Cadherin 5 in random skin flaps (200 magnification; scan bar, 25 *μ*m). (b, d) Quantification of integral absorbance of VEGF and Cadherin 5 in IHC. (e) Western blotting result of MMP9, VEGF, and Cadherin 5 in the control and CAT group. (f–h) Quantification of MMP9, VEGF, and Cadherin 5 expressions in each group. Significance: ^∗^*p* < 0.05 and ^∗∗^*p* < 0.01 vs. the control group. Data were expressed as means ± SEM, *n* = 6.

IHC and Western blotting were employed to research whether CAT improved angiogenesis in skin flaps. Here, we detected angiogenesis-related markers, including VEGF, MMP9, and Cadherin 5. Results from IHC showed that VEGF and Cadherin 5, mainly expressed in vessels and stromal cells, were upregulated significantly in the CAT group compared to the control (Figures 2(a)–2(d)). Likewise, Western blot analysis showed the increased expression of VEGF, MMP9, and Cadherin 5 due to CAT treatment (Figures 2(e)–2(h)). The findings above indicate that CAT increases skin angiogenesis, a key factor affecting flap survival.

### 3.3. CAT Reduces Oxidative Stress

**Figure 3:**
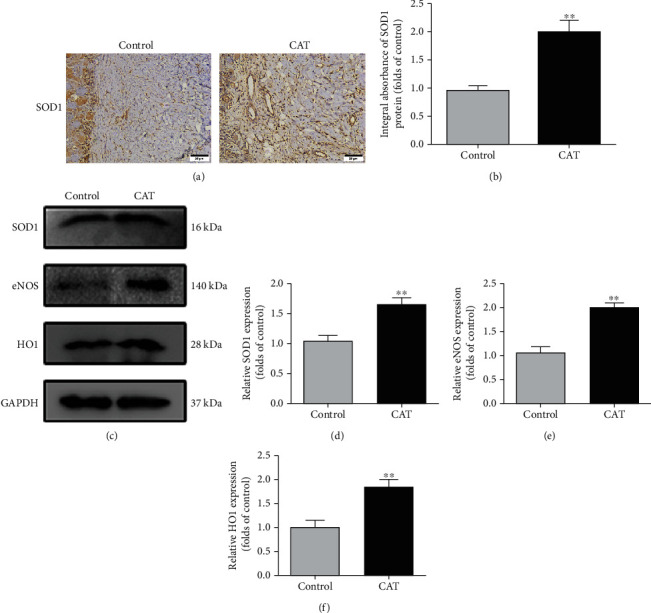
CAT alleviates oxidative stress in random skin flaps. (a) Immunohistochemical staining of proteins SOD1 in random skin flaps (200 magnification; scan bar 25 *μ*m). (b) Quantification of integral absorbance of SOD1 in IHC. (c) Western blotting result of SOD1, eNOS, and HO1 in the control and CAT group. (d–f) Quantification of SOD1, eNOS, and HO1 expressions in each group. Significance: ^∗∗^*p* < 0.01 vs. the control group. Data were expressed as means ± SEM, *n* = 6.

To further assess whether CAT could alleviate oxidative stress in an I/R flap model, we applied IHC to detect SOD1 expression, a crucial endogenous antioxidase. IHC analysis demonstrated that the integral absorbance of SOD1 was higher in the CAT group than that in the control group (Figures 3(a) and 3(b)). In addition, the status of SOD1, eNOS, and HO1 was detected by Western blotting (Figure 3(c)). The expression of these proteins was significantly increased by CAT treatment (Figures 3(d)–3(f)). Furthermore, with CAT therapy, the ROS levels were significantly reduced in flap tissues (Figure S1). Collectively, these data suggest that CAT predominately reduces oxidative stress, thus contributing to flap survival.

### 3.4. CAT Inhibits Apoptosis

**Figure 4:**
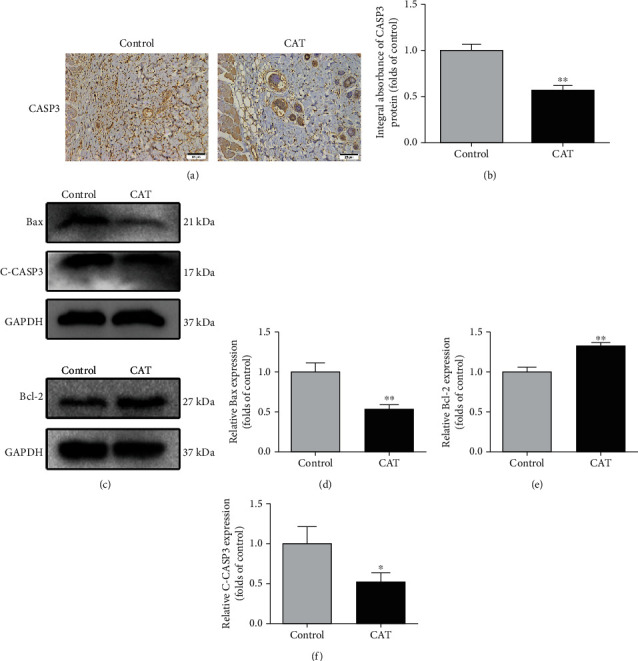
CAT inhibits apoptosis in random skin flaps. (a) Immunohistochemical staining of proteins CASP3 in random skin flaps (200 magnification; scan bar, 25 *μ*m). (b) Quantification of integral absorbance of CASP3 protein. (c) Western blotting result of Bax, Bcl-2, and C-CASP3 in the control and CAT group. (d–f) Quantification of Bax, Bcl-2, and C-CASP3 expressions in each group. Significance: ^∗^*p* < 0.05 and ^∗∗^*p* < 0.01 vs. the control group. Data were expressed as means ± SEM, *n* = 6.

To examine the protective effects of CAT on flap necrosis, the level of apoptosis was measured. As IHC has shown, the integral absorbance of CASP3 was decreased in the CAT group (Figures 4(a) and 4(b)). Similarly, Western blotting analysis showed that apoptosis-related protein Bax, Bcl-2, and C-CASP3 expressions were consistent with IHC (Figure 4(c)). The Bax and C-CASP3 protein expression levels were dramatically downregulated, while the protein expression level of Bcl-2 was upregulated (Figures 4(d)–4(f)). Together, these data suggest that CAT's beneficial effect on flap survival partly depends on inhibition of apoptosis.

### 3.5. CAT Improves Autophagy

**Figure 5:**
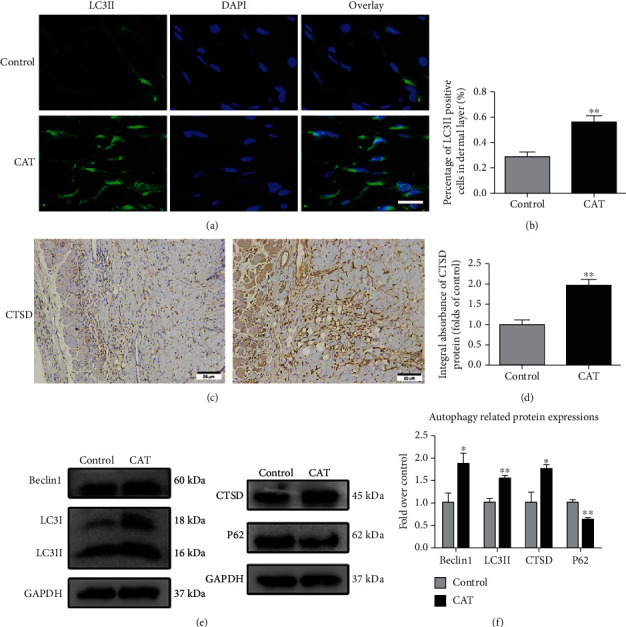
CAT improves autophagy in in random skin flaps. (a) Flap tissue sections were stained with anti-LC3II (green) immunofluorescence antibodies (DAPI staining of the nuclei). Scale bar: 10 *μ*m. (b) Quantification of percentage of LC3II positive cells in the dermal layer. (c, d) IHC and quantification analysis of CTSD in the control and CAT group. (e, f) Western blotting for expression of Belin1, LC3II, CTSD, and p62. GAPDH was used as a loading control. Significance: ^∗^*p* < 0.05 and ^∗∗^*p* < 0.01 vs. the control group. Data were expressed as means ± SEM, *n* = 6.

Based on the effects of CAT on angiogenesis, oxidative stress, and apoptosis, we hypothesized that the positive effects of CAT partly depended on autophagy. To further test the relationship between CAT and autophagy in the flap model, autophagy-related proteins were analyzed, including autophagosome component proteins Beclin1 and LC3II, lysosome marker protein CTSD, and autophagic substrate protein p62. Immunofluorescence analysis demonstrated that there were more positive cells with LC3II labeled in the dermal layer of the CAT group than the control group (Figures 5(a) and 5(b)). Furthermore, the integral absorbances of CTSD detected by IHC were enhanced in the presence of CAT (Figures 5(c) and 5(d)). As illustrated in Western blot, the expressions of Beclin1, as well as LC3II and CTSD, were increased by CAT treatment, while the expression of P62 was decreased. Taken together, these findings confirm that CAT promotes autophagy in rat random flaps.

### 3.6. Suppression of Autophagy Reverses the Positive Impacts of CAT

**Figure 6:**
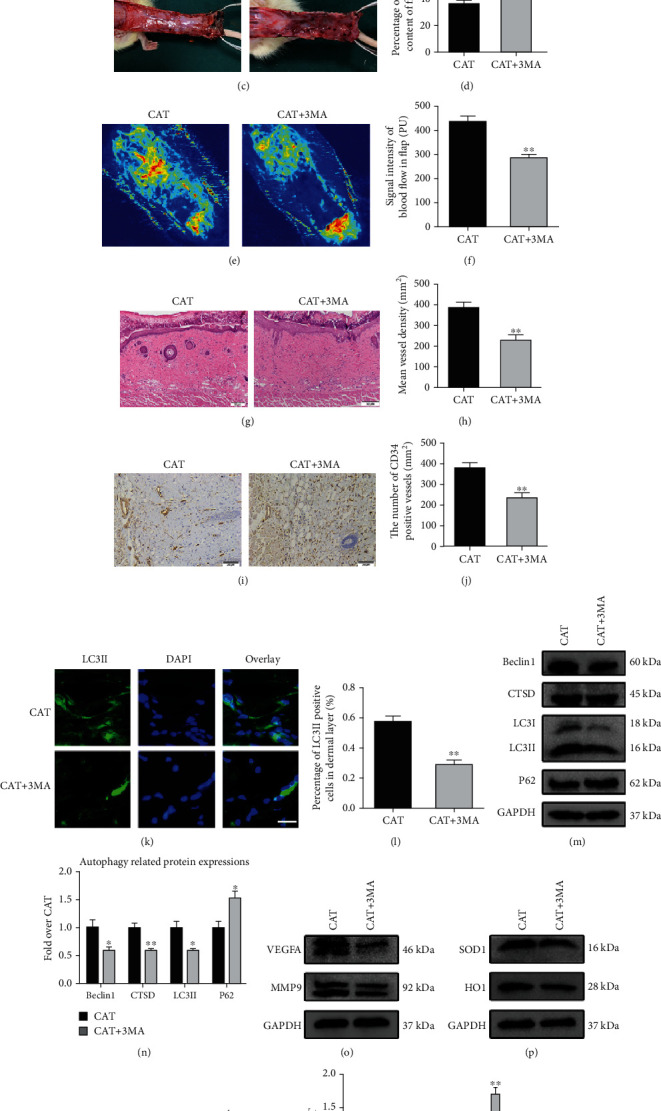
The beneficial effects of CAT on random flap viability are abolished by 3MA. (a, b) Representative images of flap necrotic area on days 3 and 7 in CAT and CAT+3MA groups after the surgery. Quantification of the percentage of survival area is shown in the histogram. (c, d) Images of the inner side of flaps in the CAT and CAT+3MA groups on day 7 after the surgery. Quantitative analysis of percentage of tissue water content in two groups. (e, f) LDBF images and quantitative analysis of flap blood flow in CAT and CAT+3MA groups. (g, h) H&E staining analysis and quantitative analysis of subcutaneous blood vessels of flaps in the CAT and CAT+3MA groups (200 magnification; scale bar, 50 *μ*m). (i, j) Immunohistochemical staining and quantitative analysis of CD34-positive vessels in CAT and CAT+3MA groups. (k, l) Immunofluorescence staining and quantitative analysis of LC3II-positive cells in dermal layer (scan bar, 10 *μ*m). (m, n) Western blotting and quantification analysis of autophagy proteins Belin1, LC3II, CTSD, and p62 in flaps after treating CAT with or without 3-MA. (o–r) Western blotting and quantification analysis of angiogenesis-related protein VEGFA and MMP9, oxidative stress-related protein SOD1 and HO1, and apoptosis-related protein Bax and Bcl-2 in each group. Significance: ^∗^*p* < 0.05 and ^∗∗^*p* < 0.01 vs. the CAT group. Data were expressed as means ± SEM, *n* = 6.

To further assess whether the regulation of autophagy is positive for flap viability, 3MA, a well-known autophagy inhibitor, was used after treatment with CAT in rat flap. In the presence of 3MA, CAT treatment could neither enhance the viability of random flap nor decrease tissue edema and subcutaneous venous congestion compared to CAT groups (Figures 6(a)–6(c)). CAT+3MA treatment yielded higher tissue water content than CAT alone. Moreover, LDBF results showed that CAT+3MA compromised blood flow compared to the CAT group (Figures 6(e) and 6(f)). As shown by HE staining, the number of microvessels in the CAT+3MA group was significantly reduced (Figures 6(g) and 6(h)). A similar finding was observed by IHC showing that the positive CD34 vessels also reduced after 3MA treatment (Figures 6(i) and 6(j)). In addition, 3MA treatment decreased LC3II-positive cells in the dermal layer in flap model (Figures 6(k) and 6(l)). Similarly, Western blot analysis showed that 3MA coadministration obviously increased the expression of p62 and significantly reduced the expression of LCII, Beclin1, and CTSD (Figures 6(m) and 6(n)). To further investigate the important role of autophagy induced by CAT for skin flap survival, we measured the regulation of CAT combined with 3MA on angiogenesis, oxidative stress, and apoptosis. Here, we found that 3MA abolished the positive effect of CAT on flap angiogenesis, oxidative stress, and apoptosis, represented by Western blot. Our results showed that 3MA reversed VEGFA and MMP9 upregulation induced by CAT treatment. Similarly, 3MA significantly reduced the expression of oxidative stress-related proteins (SOD1 and HO1). Moreover, the expression of Bax was increased, while Bcl-2 was decreased in the CAT+3MA group (Figures 6(o)–6(r)). Altogether, the above results demonstrated that autophagy was a key mechanism of CAT in the treatment of flap survival. Inhibition of autophagy by 3MA successfully abolished the effects of CAT on increasing flap survival, including increasing angiogenesis, reducing oxidative stress, and inhibiting apoptosis.

### 3.7. SIRT1 Expression Is Related to the CAT-Mediated Regulation of the Flap Survival

**Figure 7:**
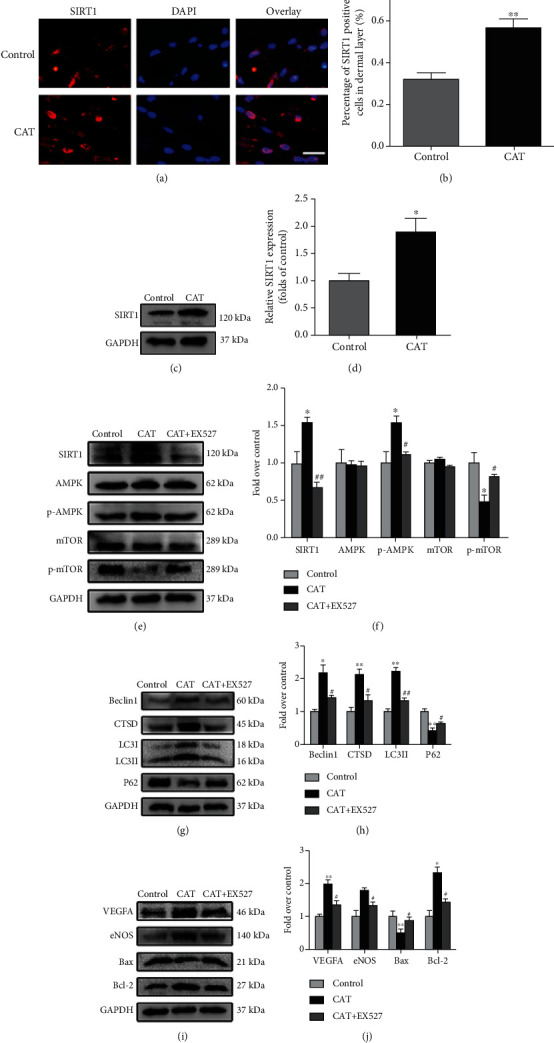
SIRT1 is involved in the protection of CAT against flap necrosis through the AMPK/mTOR signaling pathway. (a, b) Immunofluorescence staining and quantitative analysis of SIRT1-positive cells in the dermal layer in control and CAT groups after the surgery (scan bar, 10 *μ*m). (c, d) Western blotting result and quantification of SIRT1 in each group at day 7 after surgery. (e, f) Western blotting and quantification analysis of SIRT1, AMPK, p-AMPK, mTOR, and p-mTOR in the flaps from in the control, CAT, and CAT+EX527 groups. (g, h) Western blotting and quantification analysis of autophagy-related proteins Beclin1, CTSD, LC3II, and P62 in the control, CAT, and CAT+EX527 groups. (i, j) Western blotting and quantification analysis of VEGFA, eNOS, Bax, and Bcl-2 in the control, CAT, and CAT+EX527 groups. Significance: ^∗^*p* < 0.05 and ^∗∗^*p* < 0.01 vs. the control group. ^#^*p* < 0.05 and ^##^*p* < 0.01 vs. the CAT group. Data were expressed as means ± SEM, *n* = 6.

In view of SIRT1 which is a key factor in regulation of autophagy [24] and CAT which has high affinity with SIRT1, we hypothesized that CAT regulated autophagy through SIRT1. Immunofluorescence analysis showed that CAT treatment increased the SIRT1-positive cells in the flap tissue (Figures 7(a) and 7(b)). Furthermore, similar results for SIRT1 expression were found by Western blot analysis (Figures 7(c) and 7(d)). SIRT1, AMPK, and mTOR are involved in the modulation of autophagy [25]. To further clarify the role of CAT in SIRT1-related regulation of flap survival, we investigated whether AMPK-mTOR pathway was activated in random flaps treated with CAT. Our results showed that the levels of SIRT1 and phosphorylated AMPK (p-AMPK)/AMPK ratio were upregulated, while phosphorylated mTOR (p-mTOR)/mTOR ratio was downregulated in flap treated with CAT, and these effects were reversed after use of EX527, a SIRT1-specific inhibitor (Figures 7(e) and 7(f)). These data indicated that CAT activated the SIRT1-AMPK-mTOR pathway.

**Figure 8:**
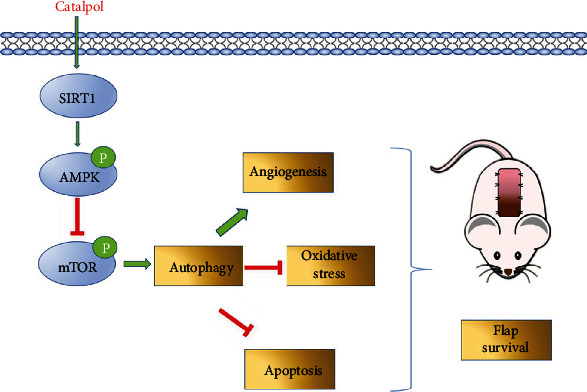
Schematic model of CAT promotes random flap survival via SIRT1-dependent autophagy mechanism. CAT increased SIRT1 expression. Overexpression of SIRT1-activated AMPL/mTOR pathway enhances autophagy to increase flap survival by increasing angiogenesis, inhibiting oxidative stress, and suppressing apoptosis.

Furthermore, we measured the effect of inhibiting SIRT1 on CAT-induced autophagy in skin flaps. Western blot analysis showed that the levels of autophagy-related proteins (Beclin1, CTSD, and LC3II) were significantly lower in the CAT+EX527 group than in the CAT group, while p62 was opposite (Figures 7(g) and 7(h)). In addition, EX527 significantly inhibited CAT-mediated upregulation of angiogenesis and suppression of apoptosis and oxidative stress in the ischemic flaps (Figures 7(i) and 7(j)). Collectively, these results illustrate that SIRT1 activation is a major mechanism by which CAT increases the level of autophagy. The regulation of the SIRT1-AMPK-mTOR pathway is at least partly involved in the protective effect of CAT in flap survival.  Figure 8 shows the schematic model of CAT.

## 4. Discussion

In this research, we demonstrate an unreported role of CAT as a SIRT1 activator in enhancing autophagy in the flap model, thus, regulating key influential factors (angiogenesis, oxidative stress, and apoptosis) for improving random flap survival. Our findings highlight CAT's protective effects against random-pattern skin flap necrosis and the important role of SIRT1 as a new target for flap therapy.

Angiogenesis after flap surgery exerts a pivotal agent in flap survival. In our research, we determined that CAT significantly increased microvascular density and CD34-positivevascular cells in the flap, thereby improving blood flow. MMP9 has been shown to exert beneficial effects on angiogenic abilities of the EPCs and play an important role in the invasion of endothelial cells and the branching of capillaries [26]. Cadherin 5 involves in controlling endothelial cell tube formation [27]. VEGF is currently being evaluated for proangiogenic and antiangiogenic therapy [28]. Our study detected that CAT treatment increased the levels of MMP9, Cadherin 5, and VEGF in flaps, indicating CAT has positive effects on angiogenesis. Consistent with the previous study that reported CAT exerted a therapeutic effect in infarcted-brain through promoting angiogenesis [29], our results demonstrated that CAT could enhance the survival of flap via improving blood supply.

I/R injury is another critical element of necrosis in the distal area of the skin flap. ROS accumulates in ischemic tissue during blood flow reperfusion, causing oxidative stress [30]. SOD, a crucial endogenous antioxidant enzyme, is beneficial to eliminate oxygen free radicals, protecting cells from I/R damage [31]. eNOS and HO1 are also known to exert antioxidant effects [32]. CAT has been shown to exert a neuroprotective effect by inhibiting oxidative stress [33]. In the current study, we found that CAT improves SOD1, eNOS, and HO1 expressions in the flap tissue and downregulates the tissue ROS accumulation, acting as a protective role against oxidative stress. Apoptosis, a commonly regulated form of cell death, could participate in I/R injury, leading to tissue necrosis. CAT has been shown to alleviate apoptosis in different diseases [34, 35]. In the random flap model, CAT could inhibit apoptosis as detected by apoptosis-related proteins Bax, Bcl-2, and C-CASP3. Taken together, our results indicate that CAT can alleviate oxidative stress and apoptosis to protect the flap from necrosis.

Autophagy is an intracellular degradation system that is critical for survival, development, and homeostasis [36]. Activation autophagy can promote the viability of random flap [37]. Previous study confirmed that CAT could attenuate hepatic steatosis by inducing autophagy [19]. To explore the mechanism of CAT in flap survival, the regulation of autophagy by CAT in the flap model was evaluated. In our present experiment, we revealed that CAT regulated the markers of autophagy activity, including increasing the levels of Beclin 1, LC3II, and CTSD, decreasing the level of SQSTM1/p62. Furthermore, the beneficial effects of CAT were inhibited after autophagy was inhibited with 3MA. In this case, angiogenesis in the flap tissue was decreased; oxidative stress and apoptosis levels were increased, resulting in flap necrosis being aggravated. Together, these results demonstrated that CAT enhanced angiogenesis and autophagy, reduced apoptosis, and alleviated oxidative stress, finally decreasing skin flap necrosis.

Given the crucial role of SIRT1 in various diseases, activation of SIRT1 may be viewed as an alternative therapeutic strategy to flap survival. Previous studies have reported positive regulation of SIRT1 in various organs, including the liver [38], lung [39], and kidney [40]. In the present study, SIRT1 expression was significantly upregulated in flap tissues after CAT treatment. Furthermore, the enhancement effects of CAT on SIRT1 were abolished, and the activities of SIRT1-regulated autophagy were inhibited, resulting in the aggravation of oxidative stress, apoptosis, and reduction of angiogenesis when the rats were coadministrated with SIRT1-specific inhibitor EX527. Collectively, we suggested that CAT could induce autophagy in random skin flaps via SIRT1 mediation to prevent flap necrosis. Mechanistically, AMPK is an important protein in the regulation of angiogenesis, oxidative stress, apoptosis, and autophagy. In addition, mTOR, as a downstream protein, decreased its phosphorylation levels when AMPK phosphorylation was increased, thus promoting autophagy [41]. Hence, our results supported the hypothesis that CAT might induce autophagy partly through the SIRT1-mediated AMPK/mTOR signaling pathway in random skin flaps, indicating the critical role of SIRT1 in flap survival and providing evidence for CAT as a potential agonist of SIRT1.

In general, new drug discoveries tend to bioactive ingredients extracted from natural sources rather than specific agonists [42]. Resveratrol, an acknowledged activator of SIRT1, has been extensively researched. However, the limitations of resveratrol are obvious due to its poor water solubility and very low bioavailability [43]. Our present study demonstrated significant activation of SIRT1 by CAT, showing favorable bioavailability in the flap model. Moreover, a previous study showed that CAT bound tightly to SIRT1 detected by docking simulations [22], making it a better choice for disease treatment.

## 5. Conclusion

In summary, our work provides evidences for the crosstalk between autophagy and SIRT1 in flaps, and the positive effects of CAT in increasing random flap survival. Our data indicate that improving SIRT1-related autophagy via CAT exerts as a potential therapeutic target for the treatment of random flap necrosis. Additionally, by applying SIRT1-specific inhibitor EX527, we verified that the regulation of the SIRT1-AMPK-mTOR pathway is at least partly involved in the positive impact of CAT.

## Data Availability

The datasets used and analyzed during the current study are available from the corresponding authors on reasonable request.

## Supplementary Materials

Supplementary Materials Fig. S1: CAT alleviates ROS levels in random skin flaps. Quantification of the ROS production in flap tissues. Significance: ^∗∗^*p* < 0.01 vs. the control group. Data were expressed as means ± SEM, *n* = 6.
