# Supplementary material for: Catalpol Enhances Random-Pattern Skin Flap Survival by Activating SIRT1-Mediated Enhancement of Autophagy
Source: Oxid Med Cell Longev. 2022 May 17;2022:5668226. doi: 10.1155/2022/5668226 (PMC9129999; doi:10.1155/2022/5668226)
Supplement: Supplementary Materials — Fig. S1: CAT alleviates ROS levels in random skin flaps. Quantification of the ROS production in flap tissues. Significance: ∗∗p < 0.01 vs. the control group. Data were expressed as means ± SEM, n = 6. [file 5668226.f1.docx]

**Supplementary material**

**Catalpol enhances random-pattern skin flap survival by activating SIRT1-mediated enhancement of autophagy**

**Ren-hao Jiang^1,2^, Chen-ji Dong^1,2^, Zhu-liu Chen^1,2^, Sheng Chen^1,2^, Jian-xin Yang^1,2^, Wei-yang Gao^1,2^***

**^1^**Department of Orthopaedics, The Second Affiliated Hospital and Yuying Children’s

Hospital of Wenzhou Medical University, Wenzhou 325027, China;

**^2^**Zhejiang Provincial Key Laboratory of Orthopaedics, Wenzhou 325027, China.

***Correspondence:**

Dr. Weiyang Gao, weiyanggaoi@126.com

^1^Department of Orthopaedics, The Second Affiliated Hospital and Yuying Children’s Hospital of Wenzhou Medical University, Wenzhou 325027, China.

^2^Zhejiang Provincial Key Laboratory of Orthopaedics, Wenzhou 325027, China.

**
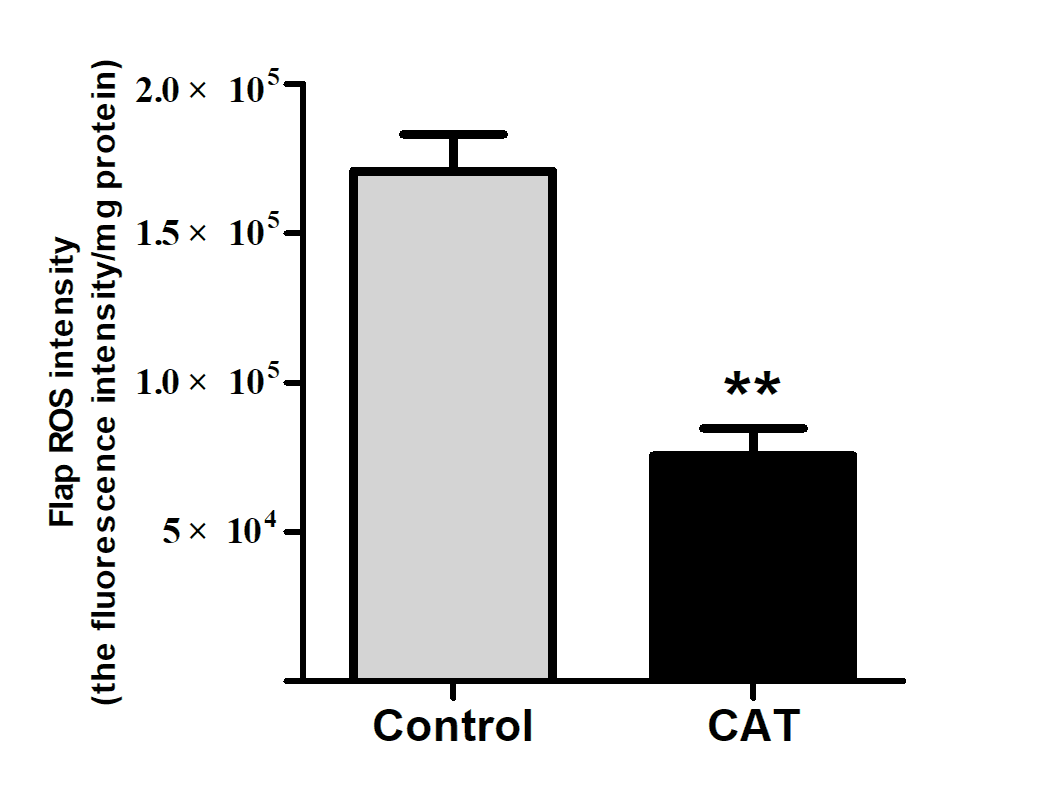
**

**Fig. S1** **CAT alleviates ROS levels in random skin flaps.** Quantification of the ROS production in flap tissues. Significance: **p < 0.01 vs. the Control group. Data were expressed as means ± SEM, n=6.
